# Possible Influence of B Chromosomes on Genes Included in Immune Response and Parasite Burden in *Apodemus flavicollis*

**DOI:** 10.1371/journal.pone.0112260

**Published:** 2014-11-05

**Authors:** Tanja Adnađević, Vladimir M. Jovanović, Jelena Blagojević, Ivana Budinski, Borislav Čabrilo, Olivera Bijelić-Čabrilo, Mladen Vujošević

**Affiliations:** 1 Department of Genetic Research, Institute for Biological Research “Siniša Stanković”, University of Belgrade, Belgrade, Serbia; 2 Department of Biology and Ecology, Faculty of Sciences, University of Novi Sad, Novi Sad, Serbia; Leibniz-Institute of Plant Genetics and Crop Plant Research (IPK), Germany

## Abstract

Genetic background underlying wild populations immune response to different parasites is still not well understood. We studied immune response to multiple infections and to competition between different parasite species at different developmental stages in population of yellow-necked mouse, *Apodemus flavicollis*. Quantitative real-time PCR was used to investigate associations of MHC II-DRB, IL-10 and Tgf-β genes expressions with presence of intestinal parasites at different developmental stages. Furthermore, we were interested whether the host related characteristics (sex, age, body condition, presence of B chromosomes or expression of other genes) or characteristics of present parasites (number of adult parasites of each identified species, egg count of each parasite genus, total number of nematode individuals) affect differential expression of the studied genes. A significant invert association between the expression of MHC II-DRB and Tgf-β gene was found, which together with absence of IL-10 association confirmed modified Th2 as the main type of immune response to nematode infections. Effect of recorded parasites and parasite life-cycle stage on expression levels of MHC II-DRB gene was detected only through interactions with host-related characteristics such as sex, age, and the presence of B chromosomes. The presence of B chromosomes is associated with lower expression level of Tgf-β gene. Although the influence of host genetic background on parasite infection has already been well documented, this is the first study in mammals that gave presence of B chromosomes on immune response full consideration.

## Introduction

In the wild, animals are constantly exposed to various parasites and pathogens. In a way, we can say that parasites are inevitable and hosts are forced to interact with many different taxa of parasites. Parasites, especially intestinal helminths, can reach high prevalence levels in their host populations influencing hosts body condition, fecundity, survival and thus fitness of individuals [1]–[3]. Due to longevity within the host, helminths have developed evolutionary coherence with their host immune system.

Mechanisms explaining host-parasite processes are mainly based on studies of laboratory rodents living under non-natural conditions that are usually pathogen-free and stress-free [4]. Laboratory models are simplified models of natural habitats with lack of constant change that exists in nature. Also, the common problem is that most interaction studies have focused on single parasite species and their host, without taking into account interspecific interactions between co-infesting parasites or the cumulative effect of co-infections [5]. However, detection of these interactions in wild rodents has been demonstrated [6], [7]. Multiple simultaneous parasite infections are almost a rule in nature [8], [9] and might exert a different pressure upon host immunity than single parasite infections [9]. Studies of naturally infected animals from wild populations can give new insights into how the immune system functions in its natural context [4], [10].

Helminth parasites induce very different immune response in comparison to bacteria, viruses, fungi, or protozoa. The immune responses of mammalian hosts are similar, despite the high diversity of helminths. To prevent or limit the damage caused by helminths, hosts have evolved a sophisticated defence system where recognition of antigens is an important step during the initiation of the immune response. In general, foreign proteins that enter cells are broken down into small peptides. The function of products of major histocompatibility complex genes (MHC) is to bind peptide fragments derived from pathogens and display them on the cell surface for recognition by the appropriate T cells. The MHC gene family encompasses two main subgroups of immunologically active molecules. Class I MHC molecules are expressed on the surface of all nucleated cells except sperm cells and some neurons, and are primarily associated with defence against intracellular pathogens such as viruses. Class II MHC molecules are expressed by antigen-presenting cells like macrophages, lymphocytes and dendritic cells. They present processed exogenous antigens to CD4+ T-helper cells. Within class II genes, majority of mammal researches focuses on the second exon of MHC-DRB genes because these loci code parts of the functionally important antigen binding sites (ABS) [11]. A subset of these protein/MHC complexes is then transported to the cell surface and presented for interrogation by the circulating T-cell population. A complex cascade of immune responses is triggered when the T cell binds to the presented peptide.

It was previously shown that potential agents of heligmosomoid nematodes that influence the host’s immunity are excretory-secretory antigens that induce regulatory T (Treg) cells *via* the transforming growth factor β (Tgf-β) signalling pathway [12]. The induced Treg cells provoke elevated levels of the immunosuppressive cytokines Tgf-β and interleukin 10 (IL-10) [13], [14] which in turn reduce MHC class II presentation in antigen presenting cells [15], [16]. These cellular changes lead to modified Th2 immune responses and parasite larvae survival [17]. It was found that the expression of regulatory cytokines like Tgf-β and IL-10 is an important feature in the course of parasitic infections because the immunological down-modulation during an infection can protect the host from pathological outcomes of the infection [14]. There is evidence that, among other important factors, the expression levels of MHC and cytokine genes play an important role in the immune response and clearance of a helminthic infection because it differs between resistant and susceptible hosts [18], [19]. While the MHC II-DRB and IL-10 genes take part only in immune processes, Tgf-β has other important biological functions beside the immune process. Tgf-β is a key player in cell proliferation, differentiation and apoptosis, and central function of Tgf-β is inhibition of cell cycle progression by regulating transcription of cell cycle regulators [20].

B chromosomes (Bs) represent additional chromosomes to the standard set of chromosomes (A set) appearing in about 15% of living species. For a long time, Bs were considered non-functional and without any essential genes, as they are dispensable for normal development and growth. But this point of view is being called into question. Influence of host genetic background on parasite infection has been well studied, but there is a deficiency of information concerning effects of B chromosomes. In spite of that, indications of the relation between Bs and parasites could be found as is the case with genes controlling resistance to rust in the B chromosomes of the common oat, *Avena sativa* [21], and the genes bringing resistance to antibiotics in the Bs of the fungus *Nectria haematococca*, favouring its pathogenicity [22], [23]. In maize, B chromosomes delay onset of leaf necrosis induced by Steak Mosaic Virus, WSMV [24]. Better resistance to parasites in the presence of odd number of Bs was observed in harvestmen, *Metagagrella tenuipes* [25].

Almost all populations of *A. flavicollis* are characterised by the presence of B chromosomes at different frequencies [26]. Previous studies in *A. flavicollis* have showed that individuals with B chromosomes respond differently to environmental changes than the ones without them, and that the presence of Bs expand populations genetic variability, allowing the species to be less vulnerable to environmental changes [27]. It has been reported that in the species *A. flavicollis*, animals with Bs have greater survival and increased fitness in habitats that are not optimal for that species [28], [29], indicating adaptive advantage of Bs carriers.

In this study we used wild-caught yellow-necked field mouse (*Apodemus flavicollis*) as non-classical model organism. The MHC class II DRB gene of *A. flavicollis* has been characterised [30] and its alleles show manifold associations to nematode susceptibility [31], [32]. The association between this gene and some parasites have been described before. *Heligmosomoides polygyrus,* is a ubiquitous helminth in this host species [33]–[35] with previously described association of infection of this parasite and lower level of MHC II-DRB gene expression [6]. Considering diversity of parasite species in wild animals, we wanted to investigate the association of expression levels of several immune genes, and present intestinal parasites, both eggs and adults, employing quantitative real-time PCR (qPCR). This is the first study which evaluates this kind of associations taking into account both eggs of a parasites and adult parasites. Furthermore, we wanted to estimate the effects of some host-related characteristics such as sex, age, body condition or genetic background (presence of B chromosomes) on expression of different immune genes. Moreover, this is the first study in mammals that considered presence of B chromosomes on immune response.

## Materials and Methods

### Sample collection

We collected 30 (10 males and 20 females) individuals in April 2012 from Mt. Avala locality using Longworth traps provided with hay and food. There were 100 traps arranged in four rows with 10 m distances between traps and between rows. The vegetation cover at sampling locality are mesophilic oak and hornbeam forest (*Querco-Carpinetum serbicum* Rudski 1949) and mesophilic beech and linden forest (*Fagetum submontanum serbicum tilietosum* Jank. et Mouse. 1954) [36]. This research was conducted under permits issued by the Ministry of natural resources, mining and spatial planning, Republic of Serbia (number: 353-03-250/2010-04). The locations sampled were not privately owned or protected in any way, and this field study did not involve endangered or protected species. The animals were treated according to Directive 2010/63/EU of the European Parliament and the Council of 22 September 2010 on the protection of animals used for scientific purposes. All animal procedures were approved by the Ethical Committee for the Use of Laboratory Animals of the Institute for Biological Research “Siniša Stanković”, University of Belgrade. Following recommendation, animals were sacrificed using ether.

Host age was determined by the dry eye lens weight [37], and host body condition index (BCI) was calculated as the residual of linear regression of total body mass on standard length [38]. Chromosomes were prepared directly from bone marrow cells using the standard technique [39]. The presence and number of Bs were determined from 30 analyzed metaphase figures. All animals with more than 48 chromosomes (standard complement) were considered to have Bs.

### Parasite burden

Collected animals were kept separated in individual cages, their mass and length were measured, and their faeces was collected and preserved in 4% formaldehyde. A survey of nematode eggs present in faeces (four samples per individual/cage) was performed by the McMaster flotation faecal egg count [40]. The intestinal tract was dissected from all of the animals and cut longitudinally in order to release its content. The contents of the intestines were deposited in water, and the supernatant was decanted until it was clear. Identification of nematodes was carried out according to the keys [41]–[43].

### RNA extraction

Thirty mg of the animal spleen tissue were used for each RNA extraction. Total RNA was extracted using TRIzol Reagent according to the manufacturer’s protocol (Life technology Inc.). Each homogenate was placed in two tubes and treated separately as independent replicates of a sample (A and B) for the subsequent procedures. Genomic DNA was digested with DNase I (Thermo Scientific). The total RNA was quantified by optical density and the quality was evaluated by gel electrophoresis. Intact rRNA subunit of 28S and 18S were observed on the gel indicating minimal degradation of the RNA.

### qPCR

Quantitative PCR was done for three target genes – MHC II-DRB gene (5′-GCGGTGACCGAGCTGGG-3′, 5′-CACAACCTCCTGGTCTGCTCT-3′), the regulatory cytokines IL-10 (5′-GGTTGCCAAGCCTTATCGGA-3′, 5′-ACCTGCTCCACTGCCTTGCT-3′) and Tgf-β (5′-TGACGTCACTGGAGTTGTACGG-3′, 5′-GCACCATCCATGACATGAACC-3′). Intron-spanning primers were used to avoid the amplification of genomic DNA contaminants. We used three reference genes (ribosomal protein S18, calnexin, and cytoplasmic γ-actin) and reference gene primers were obtained from RTPrimerDB [44] for *Apodemus flavicollis*.

We performed qPCR (Real-time PCR Prism 7000 Applied Biosystem) using Power SYBR Green RNA-to-CT 1-Step Kit (Applied Biosystems) following manufacturer’s recommendation for 10 µl volume. To avoid inter-run variation and to control RT differences we analyzed the A and B replicates of a sample in the same qPCR run. All reactions were run in triplicates (in total six reactions per individual) with a no-template control to check for contaminations. Reverse transcription was run at 48°C for 30 min. qPCR conditions were 10 min at 95°C and 40 cycles of each 95°C for 15 s, 60°C for 1 min. To confirm that only a single amplicon was present and to check for the absence of primer dimers, a melting curve analysis was performed from 60° to 95°C in 0.5°C steps lasting for 5 s. The run-specific PCR efficiency for each gene was calculated using the program 7500 System Software (Applied Biosystems).

The individual quantification cycle (Cq) was defined by the time-point when the individual fluorescence reached a threshold of 0.5 of the log-normalized fluorescence. We then calculated the individual expression value Q = E^−Cq^, where E is the mean amplification rate of each gene in each run. The arithmetic mean Q of each control gene: calnexin (Canx), ribosomal protein S18 (Rps18), and cytoplasmic γ-actin (Actg1) was calculated from the triplicate repeats and normalized to the geometric mean of the other two reference genes [45]. The arithmetic mean Q of each target gene (MHC-II DRB, IL-10 and Tgf-β) was calculated from the triplicate repeats and normalized to the geometric mean of the three reference genes [45]. These normalized relative gene expression levels were log transformed to lower the influence of the extreme values and to attribute equal weights to conditions of over- or under-expression [46]. The relative quantification of the gene of interest to reference genes allows the comparison among individuals under the assumption that the reference genes are more or less equally expressed among individuals [47]. It is still the most accurate way to detect expression differences between individuals because it controls for artificial variation, e.g. due to differences in the amount of sample, RNA extraction or reverse transcription efficiency [48].

### Data analysis

Quantitative Parasitology 3.0 software [49] was used to estimate helminth prevalence, and mean of infection intensity by bootstrapping 20,000 repeats. The prevalence is defined as the proportion of infected hosts among all the hosts examined. Mean of infection intensity is the arithmetic mean of the number of individuals of a particular parasite species per infected host in a sample. Uninfected individuals were excluded from the calculation of mean infection intensity as recommended by software manual. Afterwards, we calculated and compared observed (fobs) and expected frequencies (fexp) of parasite co-occurrence. If the occurrences of two parasites were independent from one another, the multiplied prevalence of both should give the frequency of their co-occurrence (fexp). In case of independence, the observed and expected frequencies should be the same. If observed frequency was lower than expected, we could say that there was a case of avoidance between parasites, while if observed frequency was higher than expected, there was a co-occurrence between parasites. The differences between fobs and fexp values for both egg counts and number of adult parasites were tested by two-sided Fisher’s exact test in Statistica v.5.1 [50].

In order to investigate the relationship between the expressions of studied genes and parasite load, linear models were built using different predictor variables. Explanatory variables in maximal models were host-related (sex, age, body condition index, presence of Bs in genotype, expression of other genes) and parasite-related (number of adult parasites of each identified species, egg count of each parasite genus, total number of nematode individuals). The expression rate of MHC II-DRB gene was used as a predictor in models of Tgf-β and IL-10 expression, because MHC class II genes are responsible for binding the helminth derived antigens, and triggering subsequent immune response.

The variation of each gene expression was modelled by linear least squares regression method (LM). Full models were subject to backward selection using Akaike information criterion (AIC) [51], Bayesian information criterion (BIC) [52] and log-likelihood values, in order to find the minimal best fitting model. The chosen best fitting model was the one with the lowest AIC values. All calculations and statistical tests were performed in R software package [53] with nlme 3.1–111 package [54].

## Results

The chromosome analyses showed that the frequency of animals with Bs was 0.37. The number of females with Bs was eight (frequency = 0.4) with four animals with one B and four with two B chromosomes. The number of males with Bs was three (frequency = 0.3) with two animals with one B and one with two B chromosomes. The nematode species identified from small intestine were: *Trichuris muris*, *Aonchotheca annulosa*, *Syphacia stroma*, and *Heligosomoides polygyrus*. The eggs of all identified species were present in faecal count, with the lack of distinction between the *Syphacia* species.

Overall prevalence was very high (96.7%), since only one animal did not have any parasites. Prevalence of parasite eggs was 60%, while that of adult parasites was 96.70%. Overall mean infection intensity was 2.1, parasite eggs mean infection intensity was 1.56, and adult parasites mean infection intensity was 1.90. Looking at each parasite species, the highest prevalence was detected for *H. polygyrus* (Table 1). A high prevalence was detected for *Syphacia* sp., while the other two parasite species had similar, lower prevalence. We observed a trend of higher prevalence of adult parasites than of the egg count.

**Table 1:** Prevalence and mean infection intensity of detected gastrointestinal parasites in *A. flavicollis*.

	Prevalence	Mean infection intensity
*H.polygyrus*	90	2.22
*H. polygyrus* eggs	46.7	2.43
*H.polygyrus* adults	90	2.22
*Trichuris* sp.	33.3	2.7
*Trichuris* sp. eggs	10	3.33
*Trichuris muris* adults	30	2.67
*Syphacia* sp.	53.3	2.19
*Syphacia* sp. eggs	26.7	2.38
*Syphacia stroma*	30	2.22
Capillariidae	26.7	2.88
Capillariidae eggs	20	2.83
*Aonchotheca annulosa*	26.7	2.88

The results for parasite egg count showed a significant avoidance effect between *H. polygyrus* and *Trichuris* sp. eggs (*p*<0.001), and *H. polygyrus* and *Syphacia* sp. eggs (*p*<0.001). Results for the adult parasites dataset showed significant avoidance effect between *Trichuris muris* and *Syphacia stroma* (*p*<0.001) (Table 2).

**Table 2:** Expected (fexp), observed frequencies (fobs) and their differences (diff) of single infections as well as co-occurence of detected parasites in *A. flavicollis* in egg count and adult parasites dataset separately.

Egg count	*H.polygyrus*			*Trichuris* sp.			*Syphacia* sp.			Capillariidae		
	fexp	fobs	diff	fexp	fobs	diff	fexp	fobs	diff	fexp	fobs	diff
*H.polygyrus*	0.17	0.23	−0.06									
*Trichuris* sp.	0.46	0.07	0.39***	0.04	0.03	0.00						
*Syphacia* sp.	0.74	0.10	0.64***	0.02	0.03	−0.02	0.06	0.03	0.02			
Capillariidae	0.09	0.10	−0.01	0.02	0.03	−0.01	0.03	0.07	−0.03	0.07	0.07	0.01
**Adult parasites**	***H.polygyrus***			***T. muris***			***S. stroma***			***A. annulosa***		
	**fexp**	**fobs**	**diff**	**fexp**	**fobs**	**diff**	**fexp**	**fobs**	**diff**	**fexp**	**fobs**	**diff**
*H.polygyrus*	0.18	0.16	0.02									
*T. muris*	0.33	0.30	0.03	0.07	0.04	0.03						
*S. stroma*	0.29	0.33	−0.04	0.11	0.00	0.11***	0.07	0.00	0.07			
*A. annulosa*	0.24	0.26	−0.02	0.09	0.13	−0.04	0.09	0.03	0.06	0.05	0.00	0.05

Significant differences are indicated as ***(*p*<0.001).

The best linear model for MHC II-DRB gene expression contained host sex, host age, Bs presence, *H. polygyrus* egg count, and number of *S. stroma* adults with different mutual interactions being significant (Table 3). The analysis revealed significant effect of host sex and age on MHC II-DRB expression rate. Host sex was also significant through interactions with *H. polygyrus* egg count, and with both Bs presence and number of *S. stroma* adults.

**Table 3:** Linear models explaining the effects of helminth infection intensity on studied genes expression.

Gene	Predictor	df	F value
MHC II-DRB	Sex	1	7.141*
	Age	1	7.850*
	Sex × Age	1	3.425
	Sex × Bs	2	2.327** ˙**
	Age × *S. stroma* adults	1	0.685
	Sex × *H.polygyrus* egg count	2	7.022*
	Sex × Bs × *S. stroma* adults	4	3.196**
Tgf-β	Bs	1	7.178*
	Sex	1	7.516*
	MHC II-DRB	1	10.643**

Displayed are F - statistics values. Level of significance is indicated as ***(*p*<0.001), **(*p*<0.01), *(*p*<0.05), ** ˙**(*p*<0.1).

The best linear model for Tgf-β gene expression contained Bs presence, host sex, and MHC II-DRB gene expression as predictors (Table 3). The analysis did not reveal any significant effect of helminth infections on Tgf-β gene expression. Only statistically significant factors are presented in Table 3. The analysis did not reveal any significant effects of helminth infection, or any other predictor on IL-10 gene expression.

## Discussion

Parasite species richness in host community is affected by many factors related to environmental, ecological, and evolutionary components of both host and parasite species [55]. Both species richness of detected parasites and prevalence are comparable with previous findings for population of *A. flavicollis* from Germany [35], or even higher [6]. The parasite fauna of *A. flavicollis* seems to be identical to that of *A. sylvaticus* [56] in Portugal with similar prevalence and species richness.

In their natural hosts, heligmosomoid nematodes can show a pronounced pattern of co-infection with other parasite species [4], [57], [58]. We did not observe any co-occurrence effects of studied parasite species, rather the opposite. A strong avoidance effect was observed for *H. polygyrus* eggs with *Trichuris* sp. eggs, and *Syphacia* sp. eggs, while for adults a significant avoidance effect was detected between *T. muris* and *S. stroma.* The egg avoidance effect is expected for *H*. *polygyrus* and *Syphacia* sp. since they have the same life cycle duration [59], while *Trichuris* sp. has much longer life cycle duration [60]. For the same reason, the obtained avoidance effect between *T. muris* and *S. stroma* adults was rather unexpected.

During an immune modulation process, the induced Treg cells provoke elevated levels of the immunosuppressive cytokines Tgf-β and IL-10 [13], [14], which in turn reduce the MHC class II presentation in antigen-presenting cells [15], [16]. Tregs are important in reducing pathology in a host *via* suppression of both Th1 and Th2 responses, thus preventing disease symptoms and simultaneously allowing establishment of a chronic infection. Our results showed a negative association of MHC II-DRB gene with Tgf-β and a lack of association with IL-10 gene. The study of Atlantic forest rat, *Delomys sublineatus*, found a co-expression between these three genes, which is different from our results [7]. Apart the co-expression, their PCA results indicated lower expression levels of both cytokines in contrast to high levels of MHC II-DRB gene expression. Negative association of Tgf-β gene with MHC II-DRB, in our results, was rather expected during the immune modulation process [16]. It has been suggested that during different stages or types of inflammatory responses, Treg cells require one or both of these cytokines to evoke their suppressive functions [61]. In a murine model of schistosomiasis, it was shown that IL-10 played a significant role in reducing immunopathology, especially in the chronic stage of an infection [62]. On the other hand, it has been shown that IL-10 is not required for the suppressive actions of Treg cells in helminth induced inhibition of the allergic inflammation [63].

Many factors could be associated with between-individual variations in resistance against the nematode infection. Our analysis did not reveal any significant effect of the helminth infection or any other predictor on IL-10 gene expression thus supported previous findings of dispensable role of this gene in immune modulation process.

Analysis showed sex and age as host-related variables significant for the relationship between the expressions of studied genes and parasite load. For MHC II-DRB and Tgf-β gene expression level, the analysis revealed significant effect of sex, with higher expression rates in males in general. Beside sex, the effect of age was significant but only for MHC II-DRB gene expression with higher expression in older individuals. Models with age and sex as significant factors for different aspects of host-parasite interactions in *A. sylvaticus* population were previously published [64]. It has been noted that nematode infections are usually acquired in early life and increase in intensity with age [65]. Since longevity depends on an effective immune defence against the variety of pathogens that an individual encounters over its lifetime, higher expression rate of this gene in older mice was expected since out of the studied genes, MHC II-DRB gene is the first to be expressed in an immune process.

None of the tested parasite-related variables showed a singular influence on the tested immune genes. Our results showed a significant effect of *H. polygyrus* egg count on MHC II-DRB gene expression, but only in interaction with sex. Females with *H. polygyrus* eggs had higher expression rate than males with *H. polygyrus* eggs. It was previously suggested that *H. polygyrus* influences its host’s immune system by lowering the systemic expression of MHC II-DRB gene under a natural infectious regime which can be beneficial to other parasites [6]. We have only investigated helminths, ignoring other macroparasites (arthropods) and microparasites (viruses, bacteria, fungi). The observed higher expression rate of studied genes in females with *H. polygyrus* eggs may be further linked to differential parasitic loads between the two sexes due to other parasites that were not monitored since sex-dependent trade-offs may exist between immune function components [66].

The prevailing heterochromatic nature and absence of visible phenotypic effects of Bs were the cause of their being regarded genetically inert without the claim having been tested for a long time. Although the evidence questioning this view has been accumulating slowly but constantly, only recent technological advances led to disregard of the Bs inertness paradigm. Our analysis points Bs as significant predictors for variation in expression of two out of three studied genes involved in immune response. Result showed marginal significance of effect of B chromosome presence on expressions rate of MHC II-DRB gene in interaction with sex, in such a way that males with B chromosomes had lower expression rate compared to males without B chromosomes, while females with B chromosomes had higher compared to females without B chromosomes. Also, B chromosome presence affect expressions rate of MHC II-DRB gene in interaction with sex, and the presence of *S. stroma* adults. Male mice with B chromosomes had lower, while the female ones with B chromosomes had higher expression rates. Since *H. polygyrus* and *S. stroma* have different modes of reproduction [59], it is possible that they affect MHC II-DRB gene expression at different life stages and in interaction with different host-related variables, partially independent from the immune response. The results suggest that the presence of B chromosomes could contribute to detected sex-dependant differences in MHC II-DRB gene expression.

The analysis showed that animals with B chromosomes had lower expression rate of Tgf-β gene. Although MHC II-DRB gene has a role in immune process only, Tgf-β gene has a central role in the cell cycle progression by regulating transcription of cell cycle regulators in G1 phase [20]. This could be of great importance for B chromosome which has to pass through different mitotic and meiotic check points. Tgf-β gene, through cell cycle regulation, influences development and homeostasis. The general picture of Bs obtained from molecular studies reveals that they are composed of sequences that originated from one or more A chromosomes [67]–[69]. This view of Bs suggests that the effects of B chromosomes could appear from their influence on gene regulation. Prior studies showed that three cDNA fragments were differentially expressed due to the presence of B chromosomes [70]. By elevating the expression of these genes, B chromosomes of *A. flavicollis* could affect some of the crucial processes in the cell such as tubulin and actin folding, as well as the cell metabolism. It has been suggested that there are either additional active gene(s) on Bs, or Bs might change the expression of the gene(s) on As by some trans-acting factor or by positive pleiotropic effects. Previous studies also showed higher level of morphological integration of mandible in animals with Bs in *A. flavicollis* [71]. The alveolar region was significantly more affected by the presence of Bs, with a significant increase in intensity of integration. Furthermore, Bs do not disturb developmental homeostasis in their carriers, but they play a significant role in structuring cranial variation [72]. The authors proposed that B carriers and non-carriers follow the same developmental pathways through different interactions. B carriers are characterized by direct interactions, while in mice without Bs dominant mechanism for generating covariation of cranial traits is indirect. Theoretically, integration due to parallel variation is more predisposed to modifications by natural selection which could be beneficial to B carriers under changeable environmental conditions.

In conclusion, our results show a significant invert association between MHC II-DRB and Tgf-β gene, with absence of IL-10 association, confirming modified Th2 as the main type of immune response to helminths. Animals with and without B chromosome(s) have the same endpoint immune response to parasites yet achieved through different pathways.
