# Correction: Possible Influence of B Chromosomes on Genes Included in Immune Response and Parasite Burden in *Apodemus flavicollis*

**DOI:** 10.1371/journal.pone.0118982

**Published:** 2015-04-02

**Authors:** The PLOS ONE Staff

The sixth author’s name is spelled incorrectly. The correct name is: Olivera Bjelić-Čabrilo.

There is an error in the penultimate sentence of the eighth paragraph of the Discussion. The correct sentence is: B carriers are characterized by indirect interactions, while in mice without Bs dominant mechanism for generating covariation of cranial traits is direct.
